# Flattening the Mental Health Curve: COVID-19 Stay-at-Home Orders Are Associated With Alterations in Mental Health Search Behavior in the United States

**DOI:** 10.2196/19347

**Published:** 2020-06-01

**Authors:** Nicholas C Jacobson, Damien Lekkas, George Price, Michael V Heinz, Minkeun Song, A James O’Malley, Paul J Barr

**Affiliations:** 1 Center for Technology and Behavioral Health, Geisel School of Medicine, Dartmouth College Lebanon, NH United States; 2 Department of Biomedical Data Science Geisel School of Medicine Dartmouth College Lebanon, NH United States; 3 Department of Psychiatry Geisel School of Medicine Dartmouth College Hanover, NH United States; 4 Quantitative Biomedical Sciences Program Dartmouth College Hanover, NH United States; 5 Dartmouth-Hitchcock Medical Center Lebanon, NH United States; 6 The Dartmouth Institute for Health Policy and Clinical Practice Geisel School of Medicine Dartmouth College Hanover, NH United States

**Keywords:** COVID-19, coronavirus, stay-at-home orders, mental health, suicide, anxiety, infodemiology, infoveillance, search trends, health information needs

## Abstract

**Background:**

The coronavirus disease (COVID-19) has led to dramatic changes worldwide in people’s everyday lives. To combat the pandemic, many governments have implemented social distancing, quarantine, and stay-at-home orders. There is limited research on the impact of such extreme measures on mental health.

**Objective:**

The goal of this study was to examine whether stay-at-home orders produced differential changes in mental health symptoms using internet search queries on a national scale.

**Methods:**

In the United States, individual states vary in their adoption of measures to reduce the spread of COVID-19; as of March 23, 2020, 11 of the 50 states had issued stay-at-home orders. The staggered rollout of stay-at-home measures across the United States allows us to investigate whether these measures impact mental health by exploring variations in mental health search queries across the states. This paper examines the changes in mental health search queries on Google between March 16-23, 2020, across each state and Washington, DC. Specifically, this paper examines differential changes in mental health searches based on patterns of search activity following issuance of stay-at-home orders in these states compared to all other states. The participants were all the people who searched mental health terms in Google between March 16-23. Between March 16-23, 11 states underwent stay-at-home orders to prevent the transmission of COVID-19. Outcomes included search terms measuring anxiety, depression, obsessive-compulsive, negative thoughts, irritability, fatigue, anhedonia, concentration, insomnia, and suicidal ideation.

**Results:**

Analyzing over 10 million search queries using generalized additive mixed models, the results suggested that the implementation of stay-at-home orders are associated with a significant flattening of the curve for searches for suicidal ideation, anxiety, negative thoughts, and sleep disturbances, with the most prominent flattening associated with suicidal ideation and anxiety.

**Conclusions:**

These results suggest that, despite decreased social contact, mental health search queries increased rapidly prior to the issuance of stay-at-home orders, and these changes dissipated following the announcement and enactment of these orders. Although more research is needed to examine sustained effects, these results suggest mental health symptoms were associated with an immediate leveling off following the issuance of stay-at-home orders.

## Introduction

The 2019 novel coronavirus disease (COVID-19), a symptomatically broad disease transmitted by human-to-human droplets or direct contact, has been declared by the World Health Organization to be an international crisis [1,2]. The rapid, largely uncontrolled spread of COVID-19 has impacted every facet of American life, calling for dramatic shifts in the social and professional behavior of nearly 327 million people.

In the early phase of an outbreak, reducing physical distance and interactions between individuals in a population is an effective way of stopping or limiting disease spread [3]. In early February 2020, the Centers for Disease Control and Prevention recommended social distancing across the United States as a strategy to prevent the rapid spread of COVID-19 and subsequent overburdening of the health care system [4]. Despite these and other measures, there has been an exponential spread of COVID-19. During this time, a simulation model suggested that only the most stringent social distancing interventions would be effective at reducing the spread [5]. In the absence of federal guidance, individual states have enacted varying degrees of these recommendations. Such enactment has manifested in the closure of schools and nonessential businesses and, more recently, in the issuance of shelter-in-place notices and stay-at-home orders in several states (hereafter referred to as stay-at-home orders). Stay-at-home orders reflect the most disruptive measure, resulting in a mass quarantine that restricts individuals to their place of residence except for essential travel. As of March 23, 2020, the following 11 states have issued these orders: California, Connecticut, Delaware, Illinois, Louisiana, Massachusetts, Michigan, New Jersey, New York, Ohio, and West Virginia. Of these 11 states to announce stay-at-home orders due to COVID-19, the stay-at-home orders for California, New Jersey, New York, Illinois, and Washington had taken effect as of March 23.

Although social distancing measures are necessary to protect physical health, less is known about the impact of such measures on mental health. A rapid review of the psychological impact of quarantine found that such measures were associated with high levels of psychological distress including posttraumatic stress symptoms, confusion and anger, and high prevalence of low mood and irritability [6]. The authors note that a lack of clear communication from governments to their citizens may heighten uncertainty, which could be a key driver of distress. This suggests that clear governmental action may reduce psychological distress. Nevertheless, none of the included studies in the rapid review assessed psychological distress immediately pre- and postenactment of a quarantine. Consequently, longitudinal data regarding the potential changes of mental health symptoms immediately before and after implementations of stay-at-home orders is needed.

To date, many studies across the world have employed internet search trends to study epidemiological changes in mental health [7-14]. Nearly all of these published studies have found an association between internet search behavior and real-world mental health data, although the magnitude of this effect has differed across studies. Specifically, studies have found robust associations between suicide search queries and completed suicide rates [7,10,12] with extremely high interrater reliability (intraclass correlation coefficient [ICC]=0.98) between searches using the term “suicides” and observed suicide rates [15]. Many of these studies have also found links between mental health search data and observed rates of life stressors, such as divorce and unemployment [7,10,12]. These findings provide support for using mental health queries as effective proxies for real world mental health outcomes. They in effect “bridge the gap” between internet search trends and epidemiological data, allowing for important public health application to this large corpus of readily available and easily accessible data.

This paper evaluated the impact of stay-at-home orders on mental health search queries between March 16-23, 2020. This work used Google Trends to quantify changes in search behavior in the 50 states within the United States as well as the District of Columbia (Washington, DC) with the goal of better understanding the acute mental health impact of stay-at-home orders amid COVID-19. Specifically, we sought to determine whether stay-at-home orders were associated with increased affective symptoms as might be suggested by theories related to potential impacts of prolonged social isolation, or, in contrast, whether there might be improved mental health from clear government action rather than continuing to live in a state of uncertainty caused by government inaction [16,17]. We investigated the following research questions:

1. Would stay-at-home orders significantly alter the trajectory of mental health search queries across time between March 16-23, 2020, in the United States, compared to states that had not yet enacted stay-at-home orders?
2. Would the effects of stay-at-home orders on search queries be isolated to specific symptom domains (eg, anxiety, depression, suicide) or be consistent across symptom domains?
3. Would the results of stay-at-home orders uniquely impact search queries related to mental health symptoms or be consistent with queries on physical health symptoms (both related and unrelated to COVID-19)?

## Methods

### Google Trends

**Figure 1:**
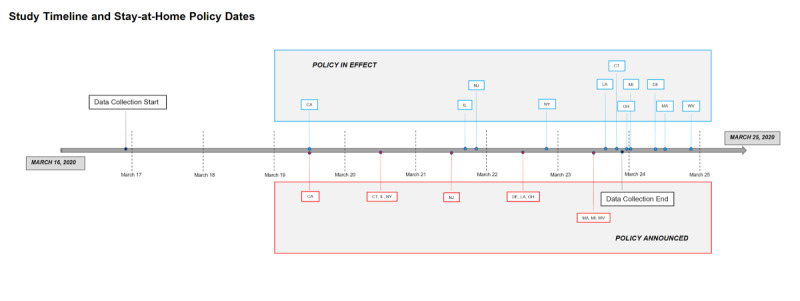
Timeline of stay-at-home order policy announcement (red) and implementation (blue).

Google is the leading search engine, retaining a dominant market share of all search traffic within the United States and worldwide. Google Trends uses large-scale search volume and allows users to download information about search volume for a given time and place. Google Trends is posted publicly, and data can be downloaded directly through Google’s web portals or through freely available software [18]. Google Trends normalizes search data per search locale (in this case, per state). Normalization occurs using the following process: each data point is divided by the total search volume of the geography and time range it represents to compare relative popularity; the results are then scaled on a range of 0-100 based on a topic’s proportion to all searches on all topics. Such normalization has the effect of controlling for the total volume of internet use across time. We used these normalized values as the outcome; a positive upward trajectory of a given search term means that term increased in relative frequency compared to other terms. The data was obtained for each hour between 11 PM on March 16, 2020, and 10 PM on March 23, 2020, EST. This period was chosen because we wanted to investigate the immediate time trends before and after issuance and implementation of stay-at-home orders. Orders were announced and implemented for 11 states within this period of time. We did not examine data prior to March 16 because Google Trends only reports hourly data for up to 7 days prior. See Figure 1 and Table 1 for a study timeline.

**Table 1:** States with stay-at-home orders as of the date of data collection.^a^

State	Policy announcement date	Policy effect date	Day difference (rounded)	Cumulative state incidence (cases), n^b^	Estimated IR^c^ (per 100,000 people)^d^
California	Thursday, March 19	Thursday, March 19	0	2267	5.7
Connecticut	Friday, March 20	Monday, March 23, 8 PM	3	415	11.6
Delaware	Sunday, March 22	Tuesday, March 24, 8 AM	2	87	8.9
Illinois	Friday, March 20	Saturday, March 21, 5 PM	1	1285	10.2
Louisiana	Sunday, March 22	Monday, March 23, 5 PM	1	1210	26.0
Massachusetts	Monday, March 23	Tuesday, March 24, noon	1	777	11.1
Michigan	Monday, March 23	Tuesday, March 24, 12:01 AM	1	1335	13.3
New Jersey	Saturday, March 21	Saturday, March 21, 9 PM	0	2844	31.8
New York	Friday, March 20	Sunday, March 22, 8 PM	2	25,665	132.0
Ohio	Sunday, March 22	Monday, March 23, 11:59 PM	1	443	3.8
West Virginia	Monday, March 23	Tuesday, March 24, 8 PM	1	22	1.2

^a^All data represented as of March 24, 2020. Policy dates were taken from examination of state news reporting as well as local government websites.

^b^Cumulative incidence (total cases) as of March 24, 2020, 1:14 PM EST [19].

^c^IR: infection rate.

^d^Total state population based on 2020 census records.

### Search Terms

#### Mental Health

We used the following search terms to examine common mental health symptoms: “anxiety,” “depression,” “OCD” (obsessive-compulsive disorder), “hopeless,” “angry,” “afraid,” “apathy,” “worthless,” “worried,” “restless,” “irritable,” “tense,” “scattered,” “tired,” “avoiding,” “procrastinate,” “insomnia,” “suicidal,” and “suicide.” These mental health terms were validated by prior research on mental health using Google Trends [20]. We also adapted other terms that measure Diagnostic and Statistical Manual of Mental Disorders, 5th edition affective disorder symptoms based on prior research assessing rapid affective symptom changes, including single-items to assess anxiety (both in terms of subjective thoughts, including fear, tense, and restless, as well as avoidance behaviors, including avoiding and procrastination), negative thoughts (hopeless, worried), irritability (anger, irritable), fatigue (tired), anhedonia (apathy), diminished ability to think or concentrate (scattered), disturbed sleep (insomnia), and suicidal ideation (suicidal, suicide) [21,22].

#### Physical Health Terms Unrelated to Known COVID-19 Symptoms

To determine whether changes in search trends were specific to searches related to mental health, we contrasted mental health symptoms to physical health symptoms that have no current known association with COVID-19. The physical health searches included: “abrasion,” “allergic,” “angina,” “apnea,” “bleeding,” “blister,” “bruising,” “conjunctivitis,” “constipation,” “discharge,” “earache,” “flatulence,” “fracture,” “hemorrhage,” “incontinence,” “inflammation,” “itching,” “lesions,” “rash,” “spasms,” “swelling,” and “syncope.” These terms were used as prespecified falsification hypotheses to provide useful control conditions to further ensure that conclusions drawn were not an artifact of the methodology alone [23].

#### Physical Health Terms Related to Known COVID-19 Symptoms

We also examined search queries for COVID-19 physical health symptoms to determine whether the changes in mental health symptoms related to stay-at-home orders were distinct from changes in COVID-19 physical health symptoms. Consequently, we also conducted searches on physical health terms among known symptoms of COVID-19 including “bloating,” “blurry,” “congestion,” “cough,” “coughing,” “croup,” “diarrhea,” “dizzy,” “fainting,” “fever,” “pain,” “sneezing,” “strep,” “stuffy,” and “vomiting.”

### Analyses

The goal of the present analyses was to investigate change in search trends across time. Consequently, it was important to account for potentially nonlinear trends across time and account for interdependence of observations. As such, the current analyses used generalized additive mixed models (GAMMs). GAMMs combine the features of models that allow predictors to have a highly flexible relationship with the outcome so long as the relationship has a smooth functional form (ie, is continuous and differentiable) such as enabled by thin plate regression splines and multilevel models that account for the lack of statistical independence in the observations made on the same units across time (see [24] for a review). Splines within GAMMs were used as they allow the data to take on any smooth functional form, but the models only allow nonlinearity in the predictor-outcome relationship if nonlinearity would provide the best fit to the data. We chose these methods over more popular but less flexible approaches (eg, higher-order polynomial transformations) because thin plate regression splines better address the issue of number and location of the knots. In particular, we made use of thin plate regression splines that use an eigenvalue decomposition to pick the basis coefficients that can explain the greatest variance. This is advantageous as it does not require a researcher to choose knot locations, thereby reducing subjectivity in modeling and otherwise having optimal bases [25], and better accommodating a higher number of predictors [26]. Notably, thin plate regression splines have been shown to not overfit the data in the way that other spline methods may [27].

We used the following model for each respective outcome:

Outcome*_i,j_* ~ s_1_(Time*_i,j_*) + s_2_(TimeSincePolicy*_i,j_*) * StayAtHomePolicy_i,j_ + *u*_1,_*_i_* + *s*_3,_*_i_*(Time*_i,j_*) **(1)**

Outcome represents each respective search term for state *i* at time *j*; Time represents the number of hours since 11 PM on March 16, 2020; TimeSincePolicy represents a time difference variable between the observed time and when the implementation of the stay-at-home policy began (defined to be 0 if the policy had not been enacted); StayAtHomePolicy represents a dummy variable representing 0 for when the policy was not in place and 1 when the policy was in place. The terms represent a smooth, thin plate regression spline, wherein the term is allowed to have a linear or nonlinear relationship with the outcome. However, nonlinearity is penalized such that data will only become nonlinear if it results in a substantially greater model fit. Note that u_1_ and s_3_ represent random effects, with u_1_ representing a vector of random intercepts of state and s_3_ representing a vector of random smooth slopes of the time trend for each state (thus allowing a nonlinear random effect to account for changes in each state). The default *k* (10) in *mgcv* value was chosen for all analyses to balance smooth fitting with computational time (as allowing a smooth spline for each random effect is computationally expensive) [28]. The latter seeks to estimate the search trajectory trend that would have occurred for that state in the absence of it implementing a stay-at-home policy. Thus, the primary term of interest is s_2_, which estimates the effects of the stay-at-home policy intervention as a deviation from the state-specific counterfactual trend that would have occurred had there not been a stay-at-home order issued.

### Ethics

This paper was not considered human subjects research because it used publicly available data, and as such was exempt from human subject approval.

## Results

### Volume of Mental Health Search Queries Across the United States

**Figure 2:**
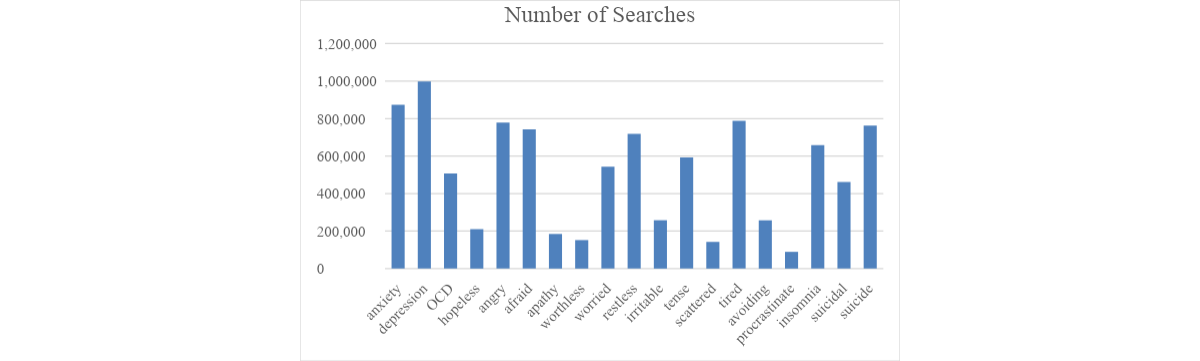
This plot depicts the estimated total volume of searches between March 18-23, 2020, for each search term. OCD: obsessive-compulsive disorder.

We estimated the total volume of search queries related to mental health by comparing and using a reported value of search terms from the Google Trends trending terms in the United States for a given day, multiplying the normalized value of each mental health term, and then dividing by the comparison term with the known absolute increase value noted across the United States. Based on this method, there were a total of 9,717,876 total searches on mental health from March 18 through March 23, 2020, (we only calculated the total search volume beginning on March 18 rather than March 16 as we were not aware of the absolute search volume terms being reported until March 25; the hour-to-hour measures reported in Google Trends only goes back a total of 7 days). See Figure 2 for a breakdown of searches per search term. Based on the average number of searches per day between March 18-23, we estimated that approximately 13 million mental health search queries were conducted between March 16-23.

### Mental Health Symptom Searches and Stay-at-Home Orders

**Figure 3:**
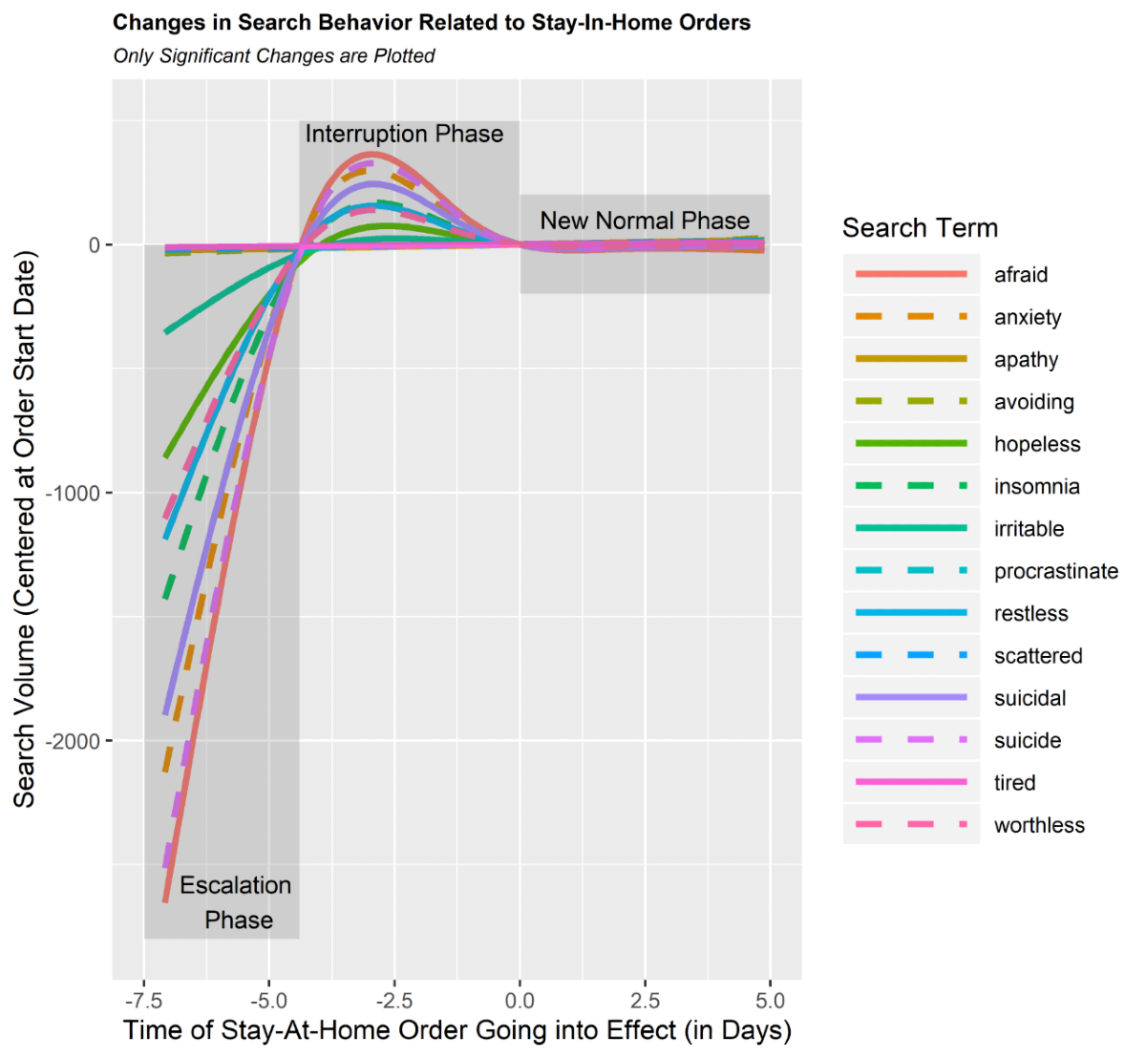
This plot depicts the changes in search patterns relative to the orders going into effect. Negative time of stay-at-home values reflects the time before the stay-at-home orders go into effect, and positive values reflect the time following the order going into effect. Note that in this plot, the predictions are not normalized to show the magnitude of the effects. The centering performed here subtracts the actual value at 0 from the estimates of this term. The escalation phase designates approximately 4 days prior to the stay-at-home orders going into effect. The interruption phase spans between approximately 4 days and 0 days before the orders go into effect, and the new normal phase represents the times the 5 days after orders go into effect.

**Figure 4:**
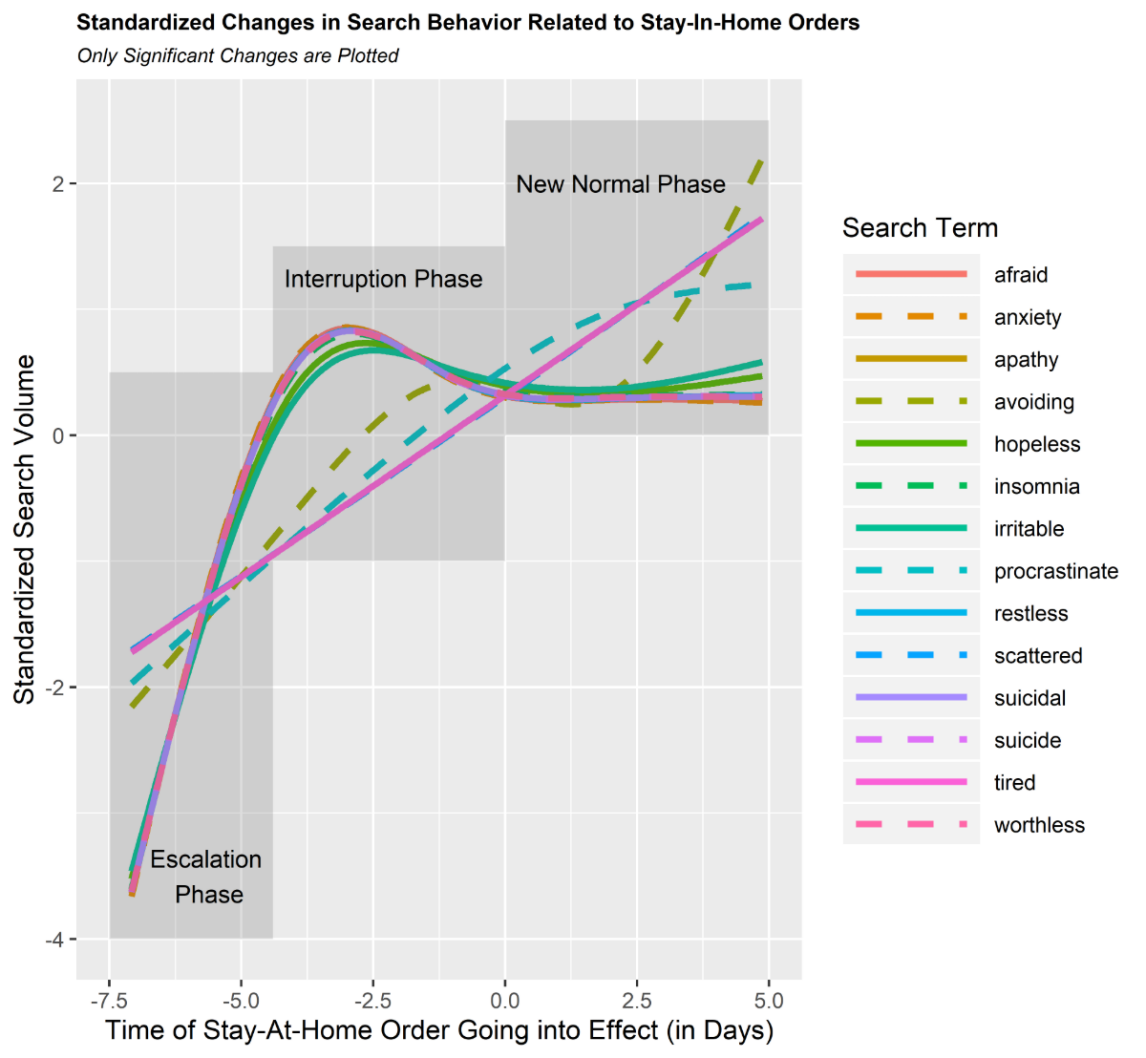
This plot depicts the standardized changes in search patterns relative to the orders going into effect. Values are normalized to reflect the relative change in these searches across time. Negative time of stay-at-home values reflect the time before the stay-at-home orders go into effect, and positive values reflect the time following the order going into effect. Values are standardized to show the relative pattern of the effect. The escalation phase designates approximately 4 days prior to the stay-at-home orders going into effect. The interruption phase spans between approximately 4 days and 0 days before the orders go into effect, and the new normal phase represents the times the 5 days after orders go into effect.

We found a significant association between queries involving 14 (of 19 total) mental health search terms and implementation of stay-at-home orders (see Table 2 for a summary). Our results showed nonlinear changes in 12 of these mental health symptoms over time associated with the announcement of stay-at-home orders. In particular, “afraid,” “anxiety,” “apathy,” “avoiding,” “hopeless,” “insomnia,” “irritable,” “procrastinate,” “restless,” “suicide,” “suicidality,” and “worthless” each showed rapid increases in search queries several days prior to the official enactment of the stay-at-home policies (see Figures 3 and 4 and Supplementary Figures A1-A3 in Multimedia Appendix 1). Moreover, each of these terms was associated with a leveling-off effect approximately 2 days prior to the implementation of the stay-at-home policy (ie, approximately the same time as the stay-at-home policy was announced). For all but 1 of these 12 symptoms (ie, “avoiding”), the leveling off remained consistent through the last day of data collection. Note that the terms associated with the strongest changes in these trends were “afraid,” “suicide,” “anxiety,” and “suicidal” (see Figure 2).

**Table 2:** Changes in search behavior related to stay-at-home orders.^a^

Search term	EDF^b^	Ref.df^c^	*F*	*P* value^d^
Anxiety	3.969	3.999	12.846	<.001
Depression	1.788	2.073	2.765	.06
OCD^e^	1.000	1.000	0.189	.66
Hopeless	3.791	3.955	14.519	<.001
Angry	2.612	2.830	1.775	.19
Afraid	4.000	4.000	16.192	<.001
Apathy	1.000	1.000	23.870	<.001
Worthless	3.888	3.985	4.349	.002
Worried	1.000	1.000	1.292	.26
Restless	3.905	3.985	4.314	.002
Irritable	3.450	3.687	4.108	.002
Tense	1.000	1.000	0.399	.53
Scattered	1.046	1.077	16.123	<.001
Tired	1.000	1.000	9.113	.003
Avoiding	2.672	2.865	5.301	<.001
Procrastinate	1.556	1.800	7.057	.005
Insomnia	3.916	3.993	10.209	<.001
Suicidal	3.970	3.999	13.446	<.001
Suicide	3.996	4.000	20.314	<.001

^a^This table corresponds to the test of the term s_2_ in the model.

^b^EDF stands for the model estimated residual degrees of freedom, where 1 corresponds to a linear deviation from the time trend.

^c^Ref.df refers to the number of model data minus the model degrees of freedom.

^d^Significant values represent the difference between what would have happened in a state with a stay-at-home policy intervention and what would have happened in the absence of that intervention.

^e^OCD: obsessive-compulsive disorder.

### Non-COVID-19 Physical Health Symptom Searches and Stay-at-Home Orders

In contrast to the mental health terms (where 14 of the 19 symptoms evidenced significant changes related to stay-at-home orders, 12 of which were associated with significant leveling off), only 7 of the 22 nonrelated physical health symptoms demonstrated changes over time related to the timing of stay-at-home orders (see Supplemental Table A1 in Multimedia Appendix 2). Of the 7, only 2 of these symptoms demonstrated a leveling-off effect (ie, flatulence and discharge). In addition, the effects of these symptoms were notably later and less extreme than those of the mental health symptoms (see Supplementary Figures A4 and A5 in Multimedia Appendix 1). Taken together, this suggests that the extreme leveling off associated with mental health symptoms was not replicated in the non-COVID-19 physical health symptoms.

### COVID-19 Physical Health Symptom Searches and Stay-at-Home Orders

Likewise, in contrast to mental health symptoms, only 6 of the 15 COVID-19 symptoms were significantly related to stay-at-home orders (see Supplemental Table A2 in Multimedia Appendix 2). Of these 6 symptoms, only 2 symptoms (fever and pain) were increasing before the initiation of the stay-at-home order, and they leveled off after the order was officially in place (see Supplementary Figures A6 and A7 in Multimedia Appendix 1). Note that the findings of the COVID-19 physical health symptoms continue to suggest that stay-at-home orders appear to more saliently impact mental health compared to COVID-19–related physical health.

## Discussion

This paper investigates the consequences of stay-at-home policies to prevent the spread of COVID-19 on changes in mental health symptom searches. The analysis is based on more than 10 million mental health Google searches within the United States. The results suggested that there were large shifts in mental health symptom searches linked to stay-at-home orders between the period of March 16-23, 2020. Particularly, the results indicated that topics related to anxiety (ie, fear, anxiety, avoiding, restlessness, procrastination), negative thoughts about oneself and the future (ie, hopelessness and worthless), sleep disturbances (ie, insomnia), and suicidal ideation (ie, suicide, suicidal) were each associated with dramatic increases prior to stay-at-home orders being announced with subsequent and considerable leveling off during these periods, approximately the same time as the stay-at-home orders were announced and enacted.

Notably, with few exceptions, these patterns were relatively unique to mental health search behaviors and not characteristic of other physical health conditions more broadly, thereby providing evidence to suggest that the largest changes were associated with mental health symptoms rather than COVID-19 symptoms across this short time frame. The consistency of these findings and their applicability to mental health domains highlights that they are likely not an artifact of the study methodology but rather reflect differences in search behaviors resulting from stay-at-home policy announcements and implementations.

Suicidality was one of the symptoms most greatly impacted by these stay-at-home orders. Importantly, prior studies have found robust associations between local Google suicide search queries and completed suicide rates [7,12], with extremely high interrater reliability (ICC=0.98) between searches for suicides and observed suicide rates [15]. Moreover, the early observed increase in “suicide” searches corroborates media reports that suicide calls have been increasing during the COVID-19 outbreak in the United States [29]. Thus, the current findings may point toward emerging evidence that stay-at-home orders may have immediately, at least temporarily, mitigated suicide risk. Although this research may point toward potential relationships between stay-at-home orders and actual suicide rates, research regarding observed suicide rates should be conducted to determine whether these patterns hold when examining actual suicide rates before any conclusions can be reached.

The current observation of rapid rises in anxiety symptom searches before the announcement of stay-at-home orders mirrors the trends of high prevalence rates of generalized anxiety disorder during the COVID-19 outbreak in China [30]. Nevertheless, the flattening of anxiety symptom searches suggest that the announcement and enactment of stay-at-home orders may have had an immediate effect on altering this rising trajectory, preventing further fears and unrest given known governmental action. Notably, the trends observed affirm prior research in China, which suggests that more clarity and action regarding precautionary COVID-19 measures leads to greater calming and lower levels of anxiety [16]. This highlights the importance of reducing uncertainty to foster mental health following disasters [24]. In turn, the societal impacts of governmental interventions in ameliorating the increase of anxiety during the COVID-19 pandemic is corroborated.

Notably, although these stay-at-home orders may have helped to flatten the staggering curvilinear increase in mental health symptom searches prior to the enactment of stay-at-home orders, the trend was not one in which these symptoms significantly decreased. Rather, the search rates remained relatively stable across the first days of a stay-at-home order going into effect. Certainly, more research will be needed to examine the long-term effects of these stay-at-home orders, especially given that longer quarantine orders have been associated with increased posttraumatic stress symptoms in prior work [31].

This study has many strengths. First, this study is the first known study to investigate the immediate impacts of stay-at-home measures during a pandemic on mental health. Second, having analyzed over 10 million search queries related to mental health across 1 week, the scope of the study methodology in examining mental health is unprecedented in size. Third, we were able to contrast the pre-post changes from these stay-at-home orders in contrast to other states in the same country to show deviations from the general pattern. Fourth, we isolated that these mental health terms were unique to mental health trajectories within the same time span by comparing the searches to changes in non-COVID-19–related and COVID-19–related physical symptoms.

Despite the study’s many strengths, the study also raises unanswered questions. Perhaps the greatest question is whether or not the flattening of these surges in mental health symptom searches is short-lived or if long-term stay-at-home orders will result in an abiding dampening of these symptoms. Thus, more research is needed to extend these findings to study the impacts of both COVID-19 and governmental responses to COVID-19 during this unprecedented time.

## Appendix

Multimedia Appendix 1 Supplementary figures.

Multimedia Appendix 2 Supplementary tables.

## Abbreviations

COVID-19: coronavirus disease
GAMM: generalized additive mixed model
ICC: intraclass correlation coefficient
OCD: obsessive-compulsive disorder
