# Supplementary material for: Flattening the Mental Health Curve: COVID-19 Stay-at-Home Orders Are Associated With Alterations in Mental Health Search Behavior in the United States
Source: JMIR Ment Health. 2020 Jun 1;7(6):e19347. doi: 10.2196/19347 (PMC7265799; doi:10.2196/19347)
Supplement: Multimedia Appendix 1 [file mental_v7i6e19347_app1.docx]

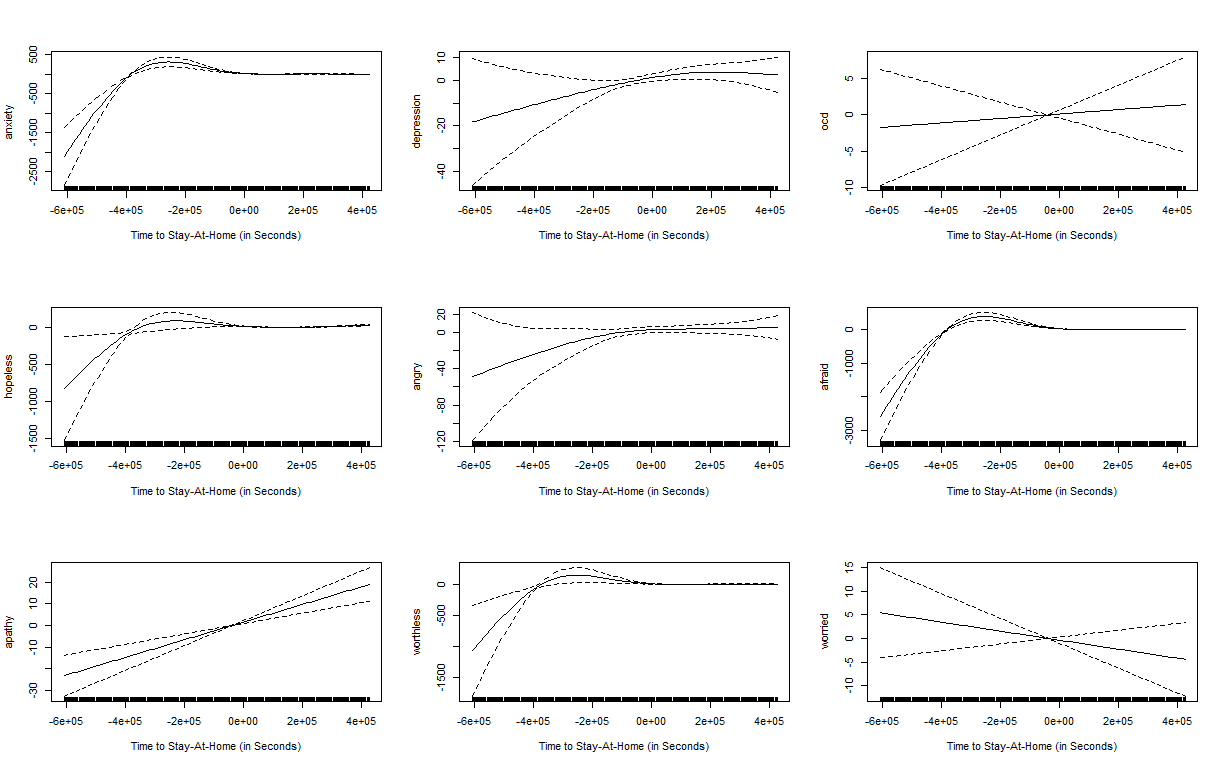


Supplemental Figure A1. This figure depicts the estimates and confidence intervals for nine of the 19 mental health symptom searches based on the stay-at-home orders (i.e. the s_2_ term).


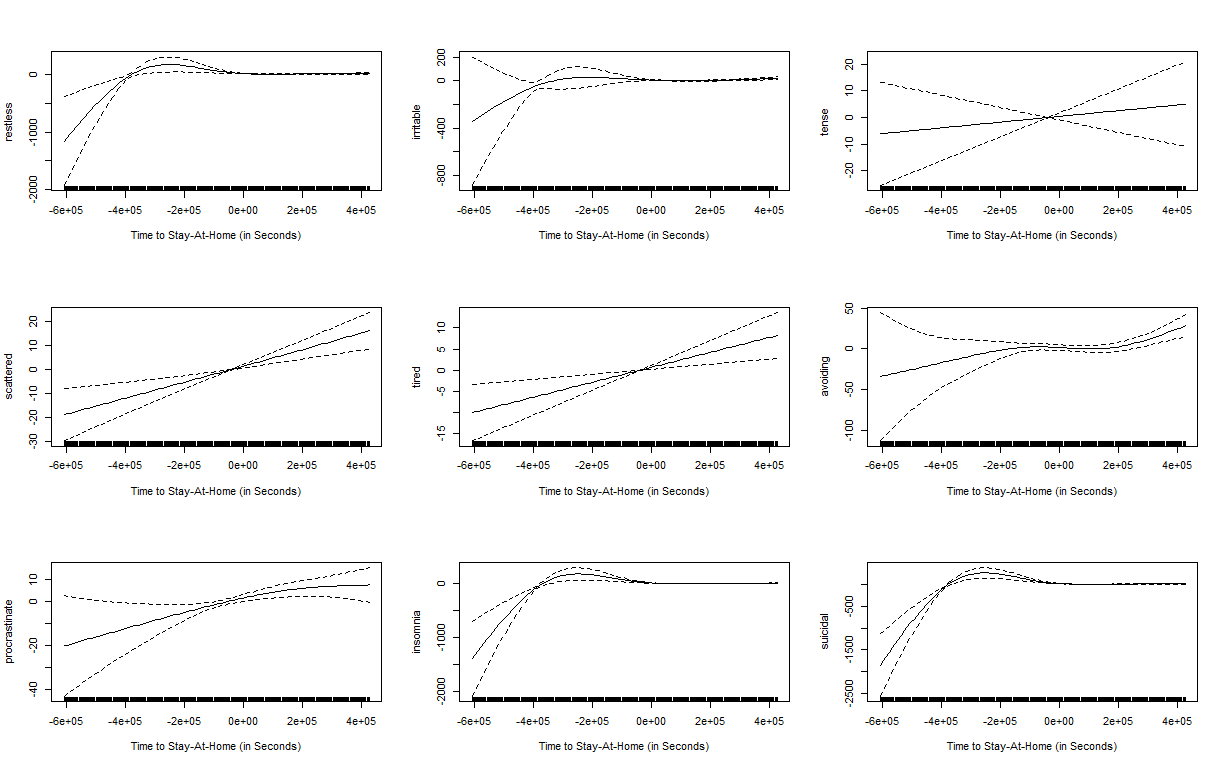


Supplemental Figure A2. This figure depicts the estimates and confidence intervals for nine of the 19 mental health symptom searches based on the stay-at-home orders (i.e. the s_2_ term).


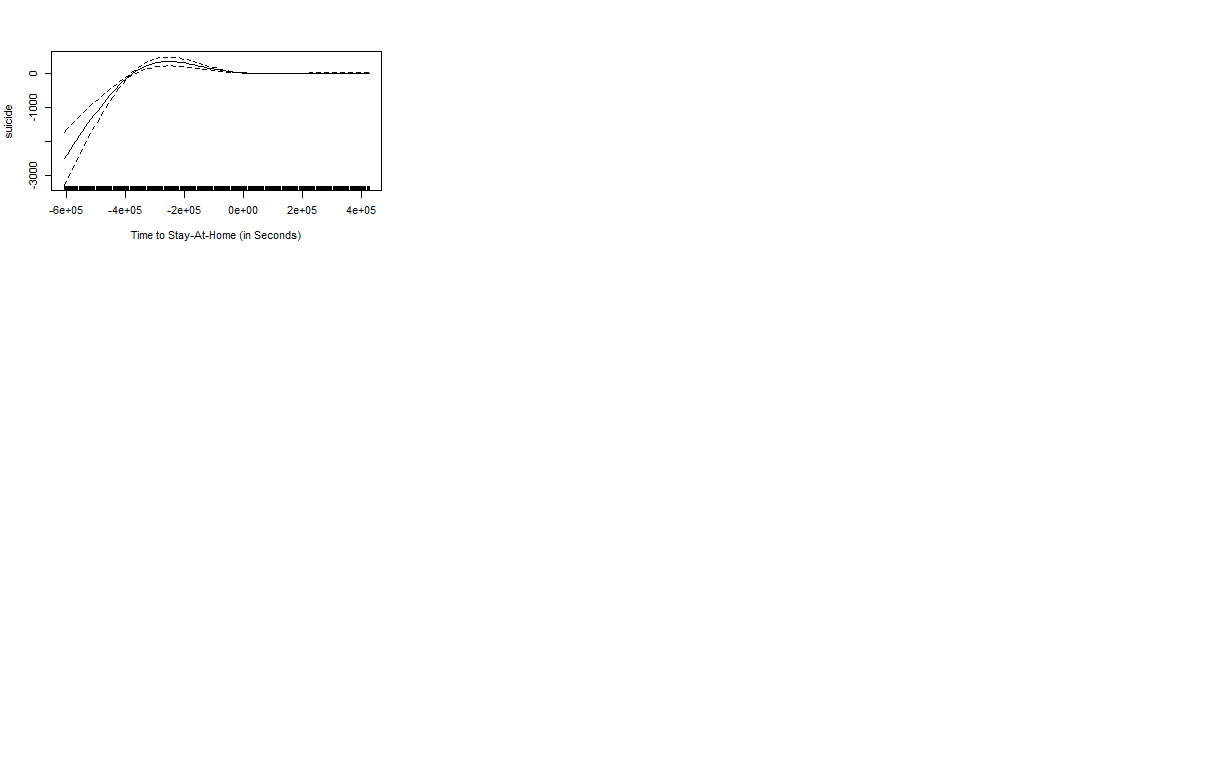


Supplemental Figure A3. This figure depicts the estimate and confidence intervals for one of the 19 mental health symptom searches based on the stay-at-home orders (i.e. the s_2_ term).


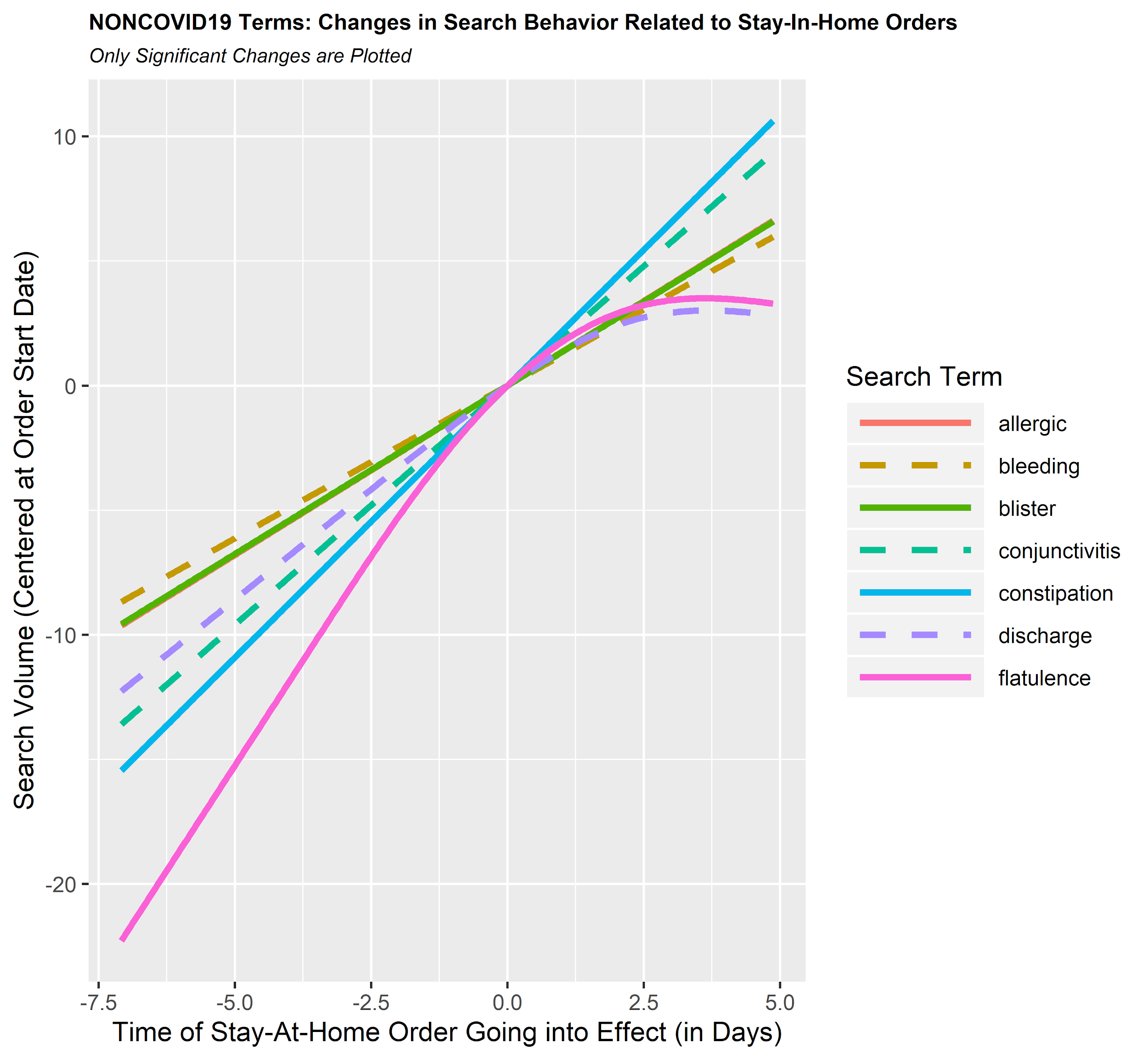
Supplemental Figure A4. This plot depicts the changes in search patterns relative to the orders going into effect. Negative time of stay-at-home values reflects the time before the stay-at-home orders go into effect, and positive values reflect the time following the order going into effect. Note that in this plot, the predictions are not normalized to show the magnitude of the effects. The centering performed here subtracts the actual value at 0 from the estimates of this term.


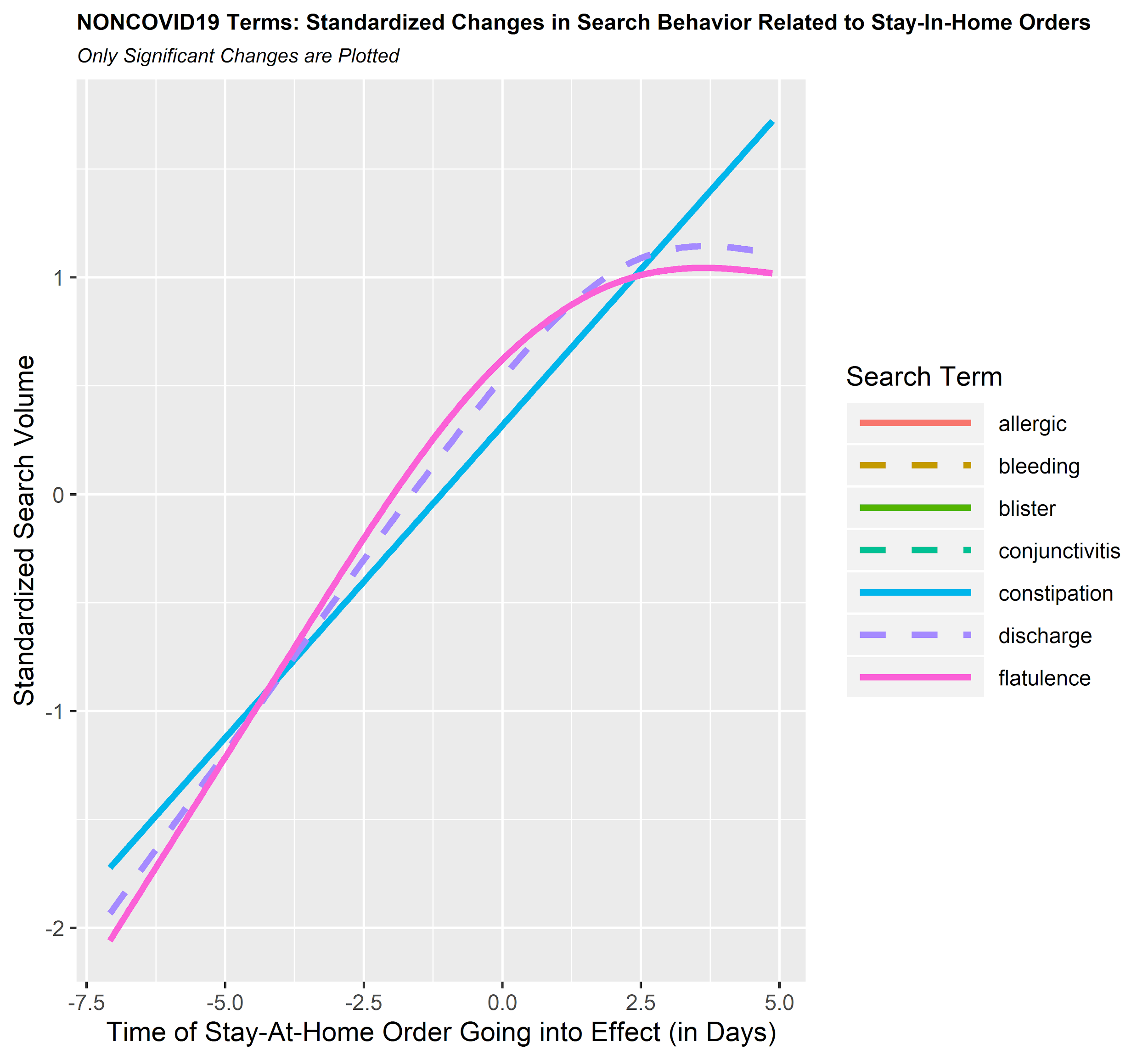


Supplemental Figure A5. This plot depicts the standardized changes in search patterns relative to the orders going into effect. Values are normalized to reflect the relative change in these searches across time. Negative time of stay-at-home values reflect the time before the stay-at-home orders go into effect, and positive values reflect the time following the order going into effect. Values are standardized to show the relative pattern of the effect.


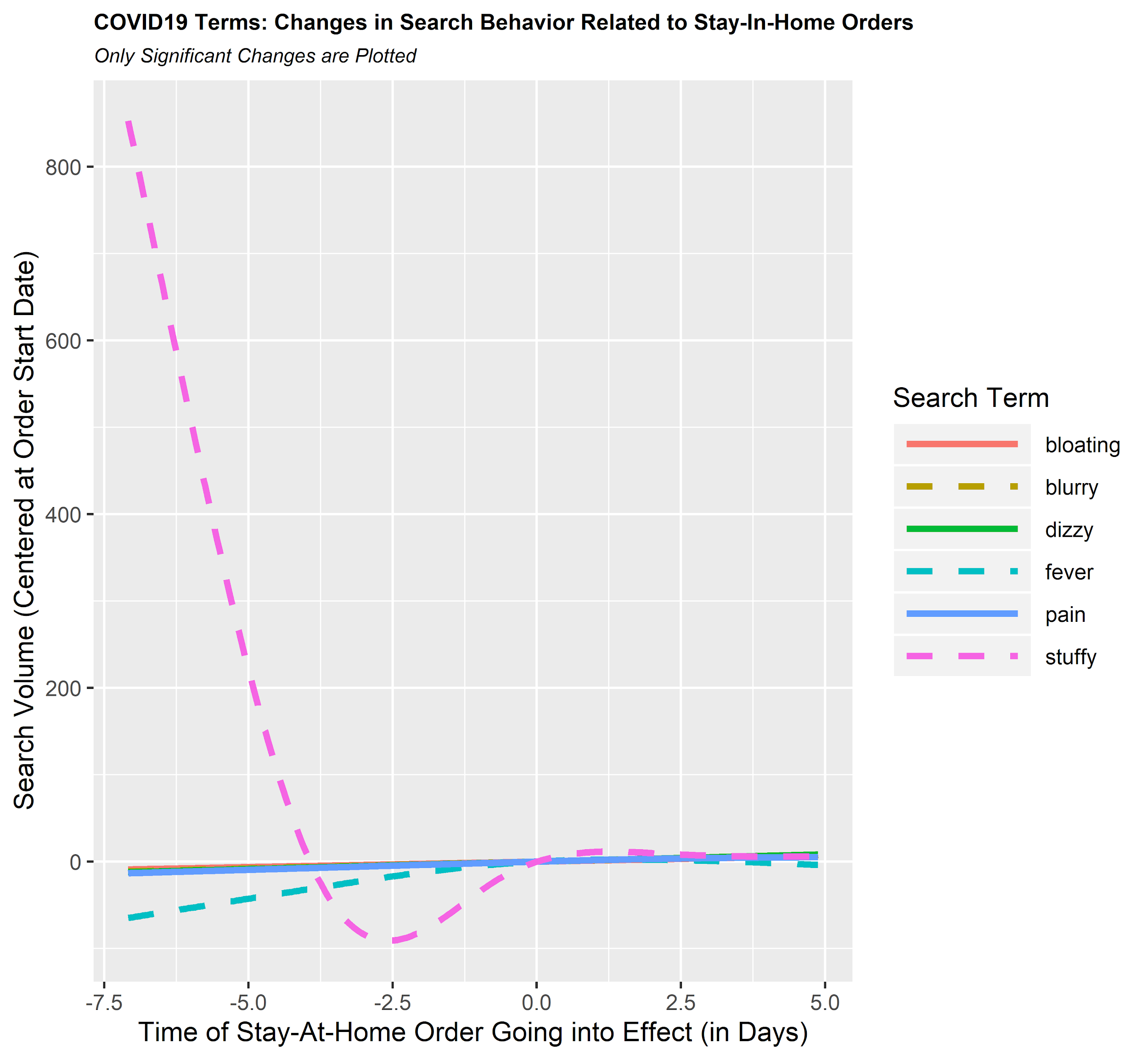


Supplemental Figure A6. This plot depicts the changes in search patterns relative to the orders going into effect. Negative time of stay-at-home values reflects the time before the stay-at-home orders go into effect, and positive values reflect the time following the order going into effect. Note that in this plot, the predictions are not normalized to show the magnitude of the effects. The centering performed here subtracts the actual value at 0 from the estimates of this term.


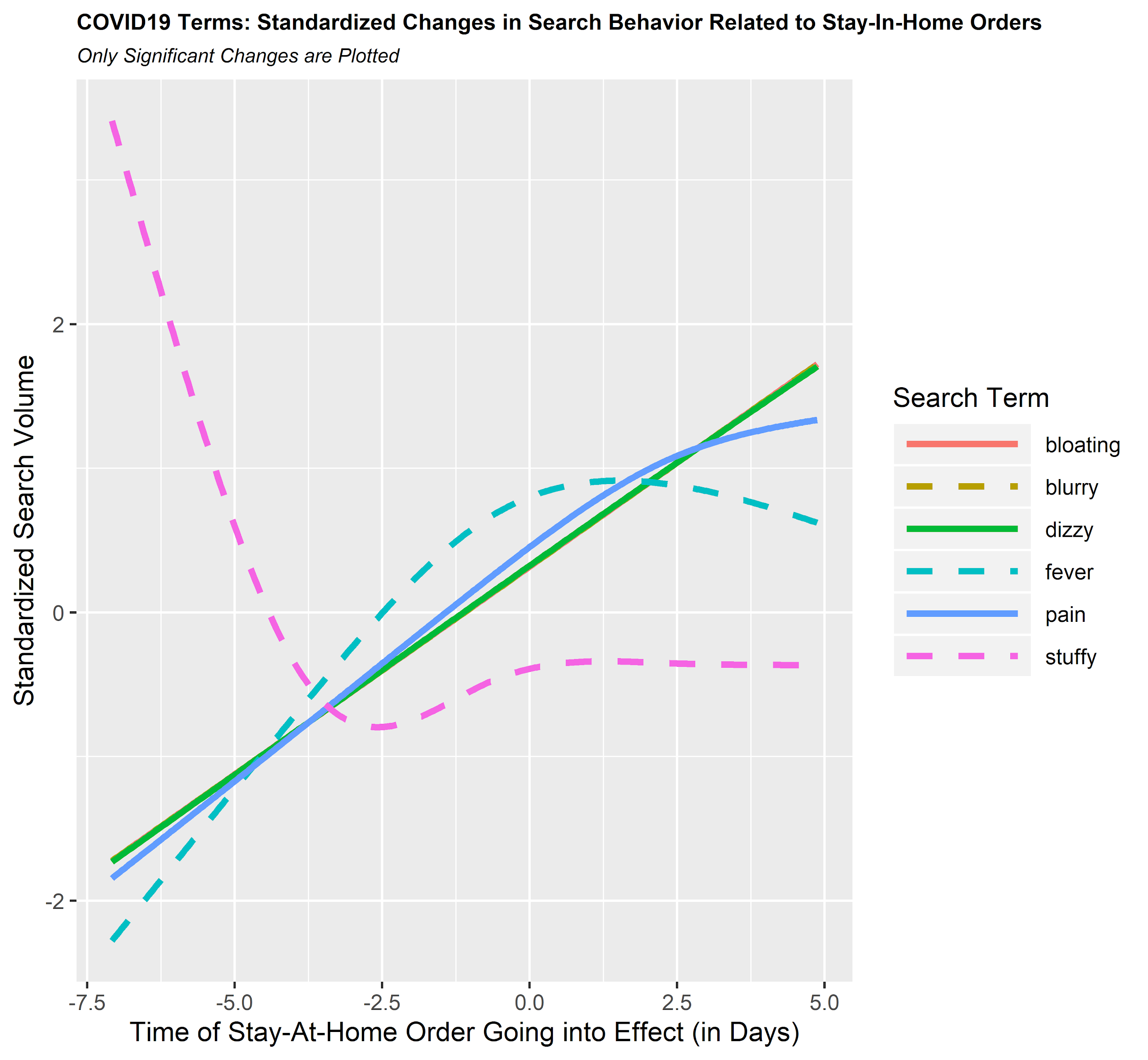


Supplemental Figure A7. This plot depicts the standardized changes in search patterns relative to the orders going into effect. Values are normalized to reflect the relative change in these searches across time. Negative time of stay-at-home values reflect the time before the stay-at-home orders go into effect, and positive values reflect the time following the order going into effect. Values are standardized to show the relative pattern of the effect.
