# Supplementary material for: Flattening the Mental Health Curve: COVID-19 Stay-at-Home Orders Are Associated With Alterations in Mental Health Search Behavior in the United States
Source: JMIR Ment Health. 2020 Jun 1;7(6):e19347. doi: 10.2196/19347 (PMC7265799; doi:10.2196/19347)
Supplement: Multimedia Appendix 2 [file mental_v7i6e19347_app2.docx]

Supplemental Table A1

Non-COVID medical terms

| **Search Term** | **edf** | **Ref.df** | **F** | **p-value** |
| --- | --- | --- | --- | --- |
| abrasion | 2.592 | 2.826 | 1.053 | 0.398 |
| allergic | 1.000 | 1.000 | 8.954 | 0.003* |
| angina | 1.304 | 1.491 | 0.742 | 0.306 |
| apnea | 1.000 | 1.001 | 0.539 | 0.463 |
| bleeding | 1.000 | 1.000 | 7.873 | 0.005* |
| blister | 1.000 | 1.000 | 4.312 | 0.038* |
| bruising | 2.503 | 2.814 | 0.415 | 0.645 |
| conjunctivitis | 1.000 | 1.000 | 11.528 | <.001* |
| constipation | 1.000 | 1.000 | 24.070 | <.001* |
| discharge | 1.630 | 1.975 | 3.633 | 0.036* |
| earache | 1.016 | 1.032 | 3.521 | 0.061 |
| flatulence | 1.781 | 2.127 | 4.310 | 0.013* |
| fracture | 2.637 | 2.850 | 2.392 | 0.149 |
| hemorrhage | 1.000 | 1.000 | 1.632 | 0.202 |
| incontinence | 1.705 | 2.049 | 2.093 | 0.130 |
| inflammation | 1.000 | 1.000 | 1.291 | 0.256 |
| itching | 1.000 | 1.000 | 1.906 | 0.167 |
| lesions | 1.000 | 1.000 | 0.003 | 0.957 |
| rash | 1.545 | 1.871 | 3.092 | 0.076 |
| spasms | 2.483 | 2.806 | 0.825 | 0.562 |
| swelling | 1.000 | 1.000 | 2.117 | 0.146 |
| syncope | 1.402 | 1.679 | 2.189 | 0.183 |

*Note*. This table corresponds to the test of the term s_2_ in the model. Significant values represent the difference between what would have happened in a state with a stay-at-home policy intervention and what would have happened in the absence of that intervention. EDF stands for the model estimated residual degrees of freedom, where 1 corresponds to a linear deviation from the time trend. Ref.df refer to the number of model data minus the model degrees of freedom.

Supplemental Table A2

COVID medical terms

| **Search Term** | **edf** | **Ref.df** | **F** | **p-value** |
| --- | --- | --- | --- | --- |
| bloating | 1.000 | 1.000 | 6.232 | 0.013* |
| blurry | 1.000 | 1.000 | 9.816 | 0.002* |
| congestion | 2.662 | 2.914 | 1.823 | 0.106 |
| cough | 1.667 | 2.018 | 0.941 | 0.380 |
| coughing | 1.445 | 1.739 | 0.985 | 0.465 |
| croup | 2.021 | 2.351 | 0.195 | 0.773 |
| diarrhea | 1.787 | 2.133 | 2.691 | 0.057 |
| dizzy | 1.027 | 1.051 | 11.276 | <.001* |
| fainting | 1.000 | 1.000 | 1.400 | 0.237 |
| fever | 2.778 | 2.934 | 7.906 | <.001* |
| pain | 1.508 | 1.823 | 5.700 | 0.010* |
| sneezing | 2.161 | 2.478 | 0.611 | 0.446 |
| strep | 3.899 | 3.989 | 2.064 | 0.069 |
| stuffy | 3.947 | 3.997 | 3.677 | 0.005* |
| vomiting | 1.000 | 1.000 | 0.293 | 0.589 |

*Note*. This table corresponds to the test of the term s_2_ in the model. Significant values represent the difference between what would have happened in a state with a stay-at-home policy intervention and what would have happened in the absence of that intervention. These contrasts are based on the differential rates of change associated with mental health symptom searches in states that enacted stay-at-home orders compared to the search rates in those same states, had they not enacted the stay-at-home-orders. EDF stands for the model estimated residual degrees of freedom, where 1 corresponds to a linear deviation from the time trend. Ref.df refers to the reference degrees of freedom (i.e. the number of observations modeled minus the model degrees of freedom).
